
JOURNAL INFORMATION
==============================
NLM Title Abbreviation: Biospektrum (Heidelb)
Journal ID: Biospektrum (Heidelb)
NLM Journal ID: 9615685

Biospektrum

ISSN: 0947-0867
EISSN: 1868-6249

ARTICLE INFORMATION
==============================
PMCID: PMC8233582
PMID: 34219979
DOI: 10.1007/s12268-021-1610-8
Article ID: 1610
Article version: 1
Subjects: Editorial

Forschungsgipfel 2021 für das deutsche Innovationssystem

Haug Gerald praesident@leopoldina.org

grid.461637.1 0000 0001 2157 0182 Deutsche Akademie der Naturforscher Leopoldina e. V., Nationale Akademie der Wissenschaften, Jägerberg 1, D-06108 Halle (Saale), Deutschland
Electronic publication date: 2021 Jun 26
Print publication date: 2021
Volume: 27
Issue: 4
First page: 345
Last page: 345
Copyright: © Der Autor 2021
License: Open Access: Dieser Artikel wird unter der Creative Commons Namensnennung 4.0 International Lizenz veröffentlicht, welche die Nutzung, Vervielfältigung, Bearbeitung, Verbreitung und Wiedergabe in jeglichem Medium und Format erlaubt, sofern Sie den/die ursprünglichen Autor(en) und die Quelle ordnungsgemäß nennen, einen Link zur Creative Commons Lizenz beifügen und angeben, ob Änderungen vorgenommen wurden. Die in diesem Artikel enthaltenen Bilder und sonstiges Drittmaterial unterliegen ebenfalls der genannten Creative Commons Lizenz, sofern sich aus der Abbildungslegende nichts anderes ergibt. Sofern das betreffende Material nicht unter der genannten Creative Commons Lizenz steht und die betreffende Handlung nicht nach gesetzlichen Vorschriften erlaubt ist, ist für die oben aufgeführten Weiterverwendungen des Materials die Einwilligung des jeweiligen Rechteinhabers einzuholen. Weitere Details zur Lizenz entnehmen Sie bitte der Lizenzinformation auf http://creativecommons.org/licenses/by/4.0/deed.de.
License URL: https://creativecommons.org/licenses/by/4.0/

issue-copyright-statement: © Springer-Verlag GmbH Deutschland, ein Teil von Springer Nature 2021

==============================
Funding note: Open Access funding enabled and organized by Projekt DEAL.

„Den Lebenswissenschaften und der Biomedizin Kommt Eine Wichtige Rolle zu: für die Biotechnologische Wertschöpfung, für den Resilienten Gesundheitsschutz und für das Erreichen Vieler Sustainable Development Goals.“

Portraitfoto: David Ausserhofer für die Leopoldina

Gerald Haug

## Forschungsgipfel 2021 für das deutsche Innovationssystem

■ Als sich mitten in der Pandemie ein Weg zur nachhaltigen Eindämmung des Coronavirus eröffnete, war dies nur dank der unerwartet schnellen Entwicklung von Impfstoffen möglich. Langfristig geförderte Grundlagenforschung, unternehmerische Initiative und risikobewusste Investitionen – diese Kombination ist die Grundlage für die gegenwärtige Hoffnung auf einen dauerhaft normalen Alltag. Damit haben Lebenswissenschaften und Biomedizin für eine breite Öffentlichkeit ihre kaum zu überschätzende Bedeutung für unser Zusammenleben prägnant bewiesen.

Nicht nur infolge dieser Krisenerfahrung ist klar, dass die Forschungs- und Innovationspolitik zwei zentrale Gestaltungsfelder für die nächste Bundesregierung sein müssen. Die Transformation unserer hochentwickelten Industriegesellschaft zu einer nachhaltigen Lebens- und Arbeitsweise – mit dem Kampf gegen den Klimawandel im Mittelpunkt – ist auf ein hervorragend funktionierendes Wissenschaftssystem in enger Verzahnung mit Innovationsprozessen in der gesamten Gesellschaft angewiesen. Innovation ohne Nachhaltigkeit ist blind und Nachhaltigkeit ohne Innovation zum Scheitern verurteilt. Darin waren sich die Expertinnen und Experten aus Wissenschaft, Wirtschaft und Politik auf dem siebten Forschungsgipfel einig, der am 19. Mai 2021 stattgefunden hat.

Seit 2015 führt der Forschungsgipfel einmal im Jahr hochrangige Persönlichkeiten aus Unternehmen, Forschungseinrichtungen, Verbänden, Exekutive und Legislative zusammen, um über drängende Fragen der Forschungs- und Innovationspolitik zu diskutieren. Üblicherweise wird er vom Stifterverband für die Deutsche Wissenschaft, der Expertenkommission Forschung und Innovation (EFI), der VolkswagenStiftung und der Nationalen Akademie der Wissenschaften Leopoldina als ganztägige Präsenzveranstaltung in Berlin mit 400 Teilnehmenden ausgerichtet. Die Coronavirus-Pandemie hat dies schon letztes Jahr verhindert, sodass nur ein virtueller Roundtable in kleiner Besetzung durchgeführt werden konnte. Auch in diesem Jahr fand der Forschungsgipfel virtuell statt, aber mit ca. 300 aus ganz Deutschland zugeschalteten Teilnehmerinnen und Teilnehmern, die sich online in die Diskussionen einbringen konnten. Viele weitere Interessenten haben sich die frei zugängliche Aufzeichnung unter www.forschungsgipfel.de angesehen.

Bundeskanzlerin Angela Merkel, Vorstandsvorsitzende großer deutscher Unternehmen sowie hochrangige Fachleute und Leopoldina-Mitglieder wie der Wirtschaftswissenschaftler Lars Feld und der Informatiker Sebastian Thrun setzten sich mit zwei Fragen auseinander: Sollte die deutsche Politik hinsichlich Forschung und Innovation stärker strategisch ausgerichtet werden, um gezielt Transformationsprozesse zu steuern? Welche Schlüsseltechnologien und Infrastrukturen müssen in Zukunft gestärkt werden, um Deutschland und Europa in der Konkurrenz mit den USA und China Handlungsspielräume zu erhalten?

Auf dem ersten Blick hat sich das deutsche Innovationssystem insgesamt rasch und flexibel an den Verlauf der Pandemie angepasst. So dürften nach Erhebungen des Stifterverbands die Aufwendungen für Forschung und Entwicklung im letzten Jahr nicht signifikant gesunken sein. Damit scheint es für die Zukunft weiterhin gut gerüstet. Doch hat die Pandemie auch Schwächen sichtbarer gemacht, die es in den kommenden Jahren zu beheben gilt, wenn Deutschland und Europa im weltweiten Wettbewerb nicht zurückfallen wollen. Gerade die Erfolge der beiden letzten Jahrzehnte Deutschlands in Wissenschaft und Innovation könnten dazu verführen, diese Schwächen zu unterschätzen.

Stattdessen müssen nach der Bundestagswahl die innovationspolitischen Weichen in Richtung auf mehr Koordination zwischen den beteiligten Ressorts und auf flexiblere Rahmenbedingungen für die Förderung vielversprechender, aber riskanter Vorhaben gestellt werden. Das schillernde Leitbild der technologischen Souveränität, das immer stärker die innovationspolitische Debatte dominiert, diskutierte der Forschungsgipfel durchaus kontrovers: Der Warnung davor, dass es leicht als Argument für die Förderung wenig zukunftsträchtiger Technologiebereiche missbraucht werden könne, stand beim Forschungsgipfel die Hoffnung gegenüber, dass es als Weckruf wirken könne, Europa wieder zum Innovationsführer bei Schlüsseltechnologien zu machen.

Den Lebenswissenschaften und der Biomedizin kommt dabei eine wichtige Rolle zu: für die biotechnologische Wertschöpfung, für den resilienten Gesundheitsschutz und für das Erreichen vieler *Sustainable Development Goals*. Die nächste große Entdeckung, den nächsten innovativen Durchbruch wird kein Forschungsgipfel voraussehen. Aber eine solche Veranstaltung kann verhindern helfen, dass beides wegen fehlender Finanzierung oder einengender Regulierung außerhalb Deutschlands oder Europas stattfindet. ■

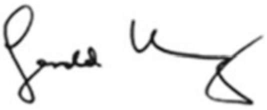

Gerald Haug
Präsident der Nationalen Akademie der Wissenschaften Leopoldina

**Funding note:** Open Access funding enabled and organized by Projekt DEAL.
**Open Access:** Dieser Artikel wird unter der Creative Commons Namensnennung 4.0 International Lizenz veröffentlicht, welche die Nutzung, Vervielfältigung, Bearbeitung, Verbreitung und Wiedergabe in jeglichem Medium und Format erlaubt, sofern Sie den/die ursprünglichen Autor(en) und die Quelle ordnungsgemäß nennen, einen Link zur Creative Commons Lizenz beifügen und angeben, ob Änderungen vorgenommen wurden. Die in diesem Artikel enthaltenen Bilder und sonstiges Drittmaterial unterliegen ebenfalls der genannten Creative Commons Lizenz, sofern sich aus der Abbildungslegende nichts anderes ergibt. Sofern das betreffende Material nicht unter der genannten Creative Commons Lizenz steht und die betreffende Handlung nicht nach gesetzlichen Vorschriften erlaubt ist, ist für die oben aufgeführten Weiterverwendungen des Materials die Einwilligung des jeweiligen Rechteinhabers einzuholen. Weitere Details zur Lizenz entnehmen Sie bitte der Lizenzinformation auf http://creativecommons.org/licenses/by/4.0/deed.de.

**Korrespondenzadresse:**
Prof. (ETHZ) Dr. Gerald Haug
Deutsche Akademie der Naturforscher
Leopoldina e. V.
– Nationale Akademie der Wissenschaften –
Jägerberg 1
D-06108 Halle (Saale)
praesident@leopoldina.org
